# Supplementary material for: Thrombin Activity in Rodent and Human Skin: Modified by Inflammation and Correlates with Innervation
Source: Biomedicines. 2022 Jun 20;10(6):1461. doi: 10.3390/biomedicines10061461 (PMC9220157; doi:10.3390/biomedicines10061461)
Supplement: Supplementary file 1 [file biomedicines-10-01461-s001.zip › biomedicines-1760817-supplementary.pdf]

Table S1. List of diseases and inflammatory markers of patients with an inflammatory cause of neuropathy.

#	Diseases	Markers	Other
SK1	Crohn's		
SK3	Fibromyalgia		
SK6	Fibromyalgia, Hashimoto's		
SK15	Psoriatic arthritis		
SK18		High CRP and ESR	
SK19			Silicone implants
SK20			Post COVID-19
SK26	Fibromyalgia		
SK31	HIV, Syphilis		
SK32	Crohn's		
SK33	Fibromyalgia, Graves, Celiac, Relapsing polychondritis		
SK34	Multiple sclerosis		
SK40			Post-vaccination syndrome
SK45	Hashimoto's Thyroiditis		
SK46		High CRP and ESR	
SK47			Post-vaccination syndrome
SK48		High CRP	
SK51	Psoriatic arthritis		
SK55	Endometriosis		

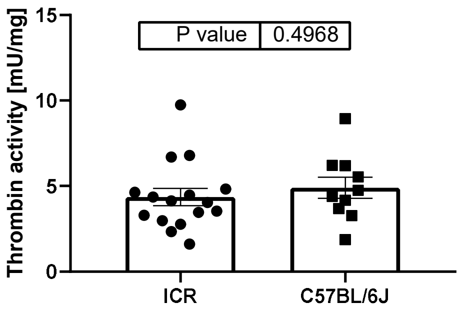

Figure S1. Thrombin activity in the skin of ICR VS C57BL/6J mice: no significant difference was found in skin thrombin activity between the two strains.
